# Delayed skin reaction after mRNA-1273 vaccine against SARS-CoV-2: a rare clinical reaction

**DOI:** 10.1186/s40001-021-00557-z

**Published:** 2021-08-25

**Authors:** Norman-Philipp Hoff, Noemi F. Freise, Albrecht G. Schmidt, Parnian Firouzi-Memarpuri, Julia Reifenberger, Tom Luedde, Edwin Bölke, Stephan Meller, Bernhard Homey, Torsten Feldt, Björn Erik Ole Jensen, Verena Keitel, Livia Schmidt, Kitti Maas, Jan Haussmann, Balint Tamaskovics, Wilfried Budach, Johannes C. Fischer, Bettina Alexandra Buhren, Wolfram Trudo Knoefel, Marion Schneider, Peter Arne Gerber, Alessia Pedoto, Dieter Häussinger, Olaf Grebe, Martijn van Griensven, Stephan A. Braun, Stefan Salzmann, Amir Rezazadeh, Christiane Matuschek

**Affiliations:** 1 Department of Dermatology, University Hospital Düsseldorf, Medical Faculty, Heinrich-Heine-University, 40225 Düsseldorf, Germany; 2 Department of Gastroenterology, Hepatology and Infectious Diseases, University Hospital Düsseldorf, Medical Faculty, Heinrich Heine University, Düsseldorf, Germany; 3 Department of Radiation Oncology, University Hospital Medical Faculty, Heinrich-Heine-University, Moorenstr. 5, 40225 Düsseldorf, Germany; 4 Institute for Transplant Diagnostics and Cell Therapeutics, Heinrich Heine University, Düsseldorf, Germany; 5 Medical Faculty, Heinrich-Heine-University, 40225 Düsseldorf, Germany; 6 Department of Surgery and Interdisciplinary Surgical Intensive Care Unit Medical Faculty, Heinrich Heine University, Düsseldorf, Germany; 7 Division of Experimental Anesthesiology, University Hospital Ulm, Ulm, Germany; 8 Department of Anesthesiology, Memorial Sloan Kettering Cancer Center, New York, USA; 9 Department of Cardiology and Rhythmology, Petrus Hospital, Wuppertal, Germany; 10 Department cBITE, MERLN Institute for Technology-Inspired Regenerative Medicine, Maastricht University, Maastricht, The Netherlands; 11 Department of Dermatology, University Hospital Münster, Münster, Germany

**Keywords:** Inflammation, COVID-19, Dermatitis, Erythema, Edema

## Abstract

**Background:**

The coronavirus disease 2019 (COVID‐19) is associated with a wide clinical spectrum of skin manifestations, including urticarial, vesicular, vasculitic and chilblain‐like lesions. Recently, delayed skin reactions have been reported in 1% individuals following mRNA vaccination against SARS-CoV-2. The exact pathophysiology and the risk factors still remain unclear.

**Patients and methods:**

6821 employees and patients were vaccinated at our institutions between February and June 2021. Every patient received two doses of the mRNA-1273 vaccine in our hospitals, and reported back in case of any side effects which were collected in our hospital managed database.

**Results:**

Eleven of 6821 vaccinated patients (0.16%) developed delayed skin reactions after either the first or second dose of the mRNA-1273 vaccine against SARS-CoV-2. Eight of 11 patients (73%) developed a rash after the first dose, while in 3/11 (27%), the rash occurred after the second dose. More females (9/11) were affected. Four of 11 patients required antihistamines, with two needing additional topical steroids. All the cutaneous manifestations resolved within 14 days. None of the skin reactions after the first dose of the vaccine prevented the administration of the second dose. There were no long-term cutaneous sequelae in any of the affected individuals.

**Conclusion:**

Our data suggests that skin reactions after the use of mRNA-1273 vaccine against SARS-CoV-2 are possible, but rare. Further studies need to be done to understand the pathophysiology of these lesions.

## Introduction

The extended vaccination campaign started in 2020 against SARS-CoV-2 infections has contributed to a significant decrease in the number of infected symptomatic patients. Novel mRNA-based vaccines have been developed, tested and made available to the world population at an unprecedented pace. While it is demonstrated that full vaccination protects against COVID-19 infection, it is still unclear how to identify and treat its unexpected side effects [1]. Baden et al. reported an 84.2% rate of immediate injection-site reactions after the first mRNA-1273 vaccination dose in their phase III trial participants [2]. Late injection site reactions occurred in 2444/30420 (8%) patients after 8 days from the first dose, and in 68/30420 (0.2%) cases after the second dose [2].

These reactions involved erythema, induration and soreness, which typically resolved within 4–5 days. Based on histological findings, the research group interpreted the side effect as a type IV hypersensitivity reaction [3–5]. Similar skin reactions have recently been reported with BNT162b2, the second currently approved mRNA-based vaccine [6, 7]. The US Centers for Disease Control and Prevention (CDC) refers to these rare skin reactions associated with mRNA-based vaccines as “COVID arm” [8]. “COVID arm” usually neither requires treatment nor should discourage a second dose of vaccination if scheduled.

Here, we report a case series of 11 patients with skin reactions after the inoculation of either the first or the second dose of the mRNA-1273 vaccine. All the reported lesions were near the injection site and presented after the complete resolution of the initial local and systemic symptoms associated with the vaccination. In addition, we describe the therapeutic options and speculate on the possible pathophysiology, based on histological examinations.

## Patients and methods

After the roll out of a massive vaccination campaign at our institutions, we collected data of vaccination and side effects in 6821 patients and health care workers who presented for their two doses between January and June of 2021. All patients received the mRNA-1273 vaccination at the recommended time intervals. The data were collected in our institutional patient database. Patients with cutaneous side effects were identified though the database after a query for cutaneous reaction. We analyzed the reports on acute side effects in the Departments of Dermatology at the University Düsseldorf and Münster  and in one private practice in Düsseldorf. Every patient providing informed consent was included in this case series. Eleven patients were identified with the side effects of interest for our study during our vaccination program. These patients returned to the Dermatology Department or the Department of Infectious Disease to address the acute side effects after the vaccination dose.

## Results

The 11 patients are individually described below. A summary of their data is provided in Table 1.

**Table 1:** Case description of the 11 patients who developed a skin reaction after COVID vaccination

Case	Age (years)	Sex	Comorbidity	First/second injection	Time onset of the skin reaction (days)	Treatment	Relief of symptoms (days)
1	56	Male	No	First	3	Oral antihistamines	1
2	60	Female	No	First	4	Oral antihistamines	1
3	41	Female	No	First	7	No	2
4	41	Female	No	First	7	No	2–3
5	50	Female	No	First	9	No	2–3
6	30	Female	No	First	7	Topical glucocorticoids + oral antihistamines	3–4
7	44	Female	Obesity	Second	3	No	2
8	63	Female	No	Second	2	No	2
9	50	Female	No	Second	4	Topical glucocorticoids + oral antihistamines	
10	37	Female	No	First	8	No	3
11	79	Male	No	First	12	No	4

### Case 1

**Fig. 1:**
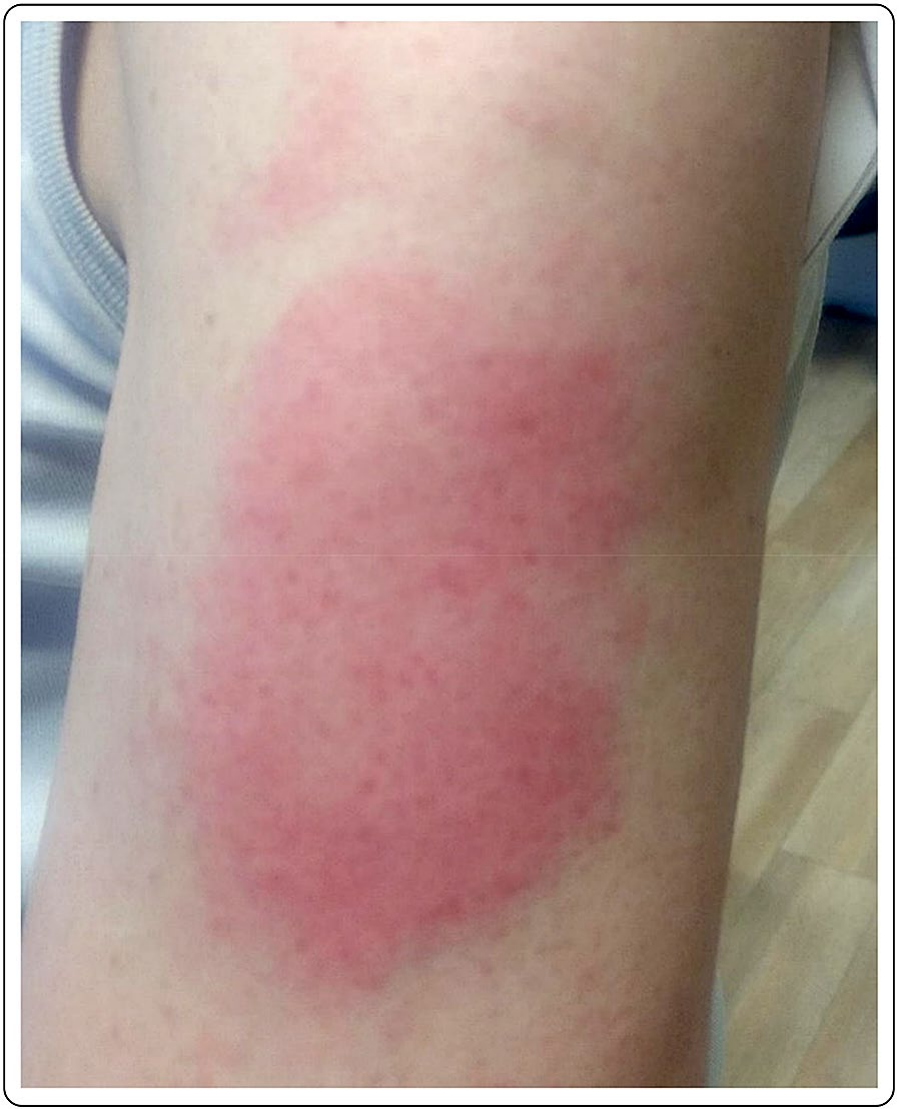
“COVID arm”: delayed cutaneous reaction to the mRNA-1273 vaccine, with erythema and induration 72 h after the injection in a 56-year-old male

A 56-year-old Caucasian male with no past medical history presented with a large area of local erythema and edema at the injection site, 3 days after the first dose of the mRNA-1273 vaccine, in addition to local cutaneous hypersensitivity. The use of oral antihistamines quickly alleviated his symptoms (Fig. 1).

### Case 2

**Fig. 2:**
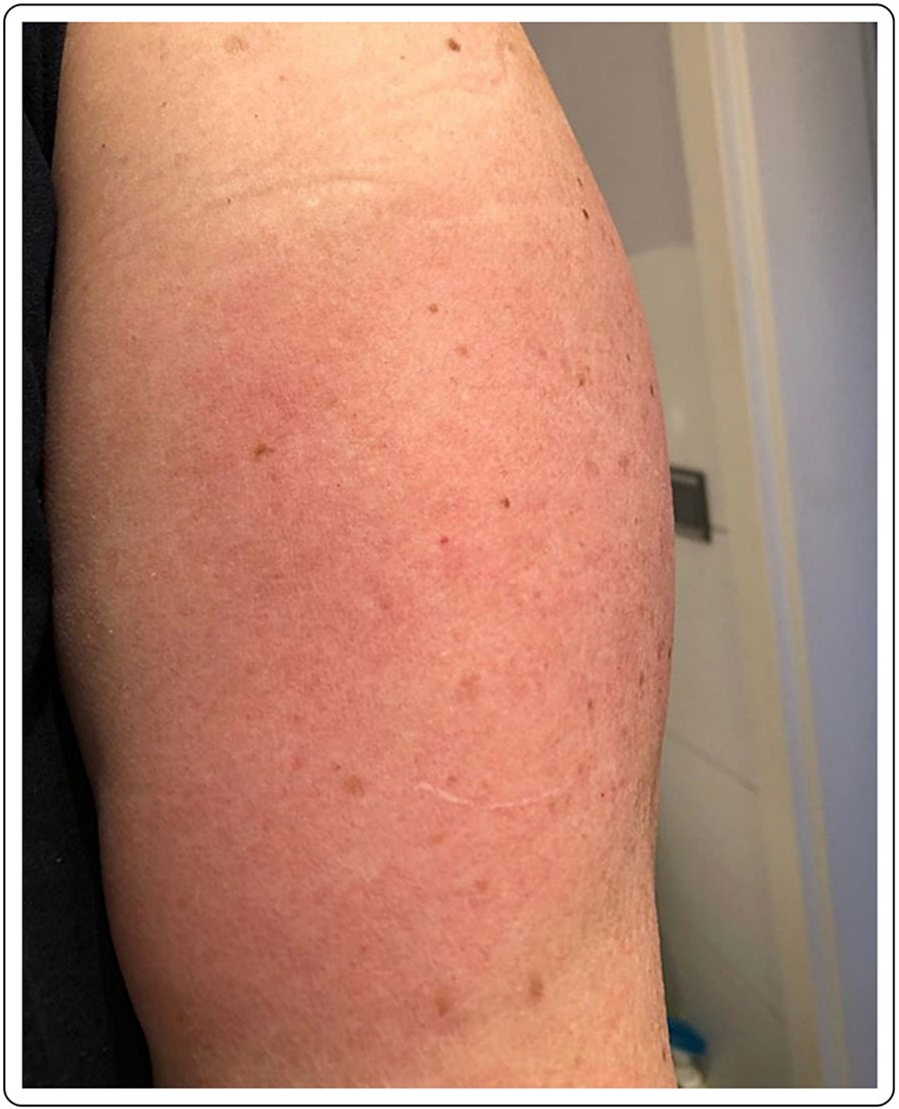
“COVID arm”: 48 h after the injection with the mRNA-1273 vaccine, large area of local erythema and edema at the injection site, in conjunction with cervical lymphadenopathy, 4 days after the first dose

A 60-year-old Caucasian female with no past medical history presented with a large area of local erythema and edema, which developed at the site of the injection 4 days after the first dose of the mRNA-1273 vaccine. Cervical lymphadenopathy was also present starting 2 days after the first injection. Oral antihistamines were useful for the symptoms, which did not present after the second dose (Fig. 2).

### Case 3

**Fig. 3:**
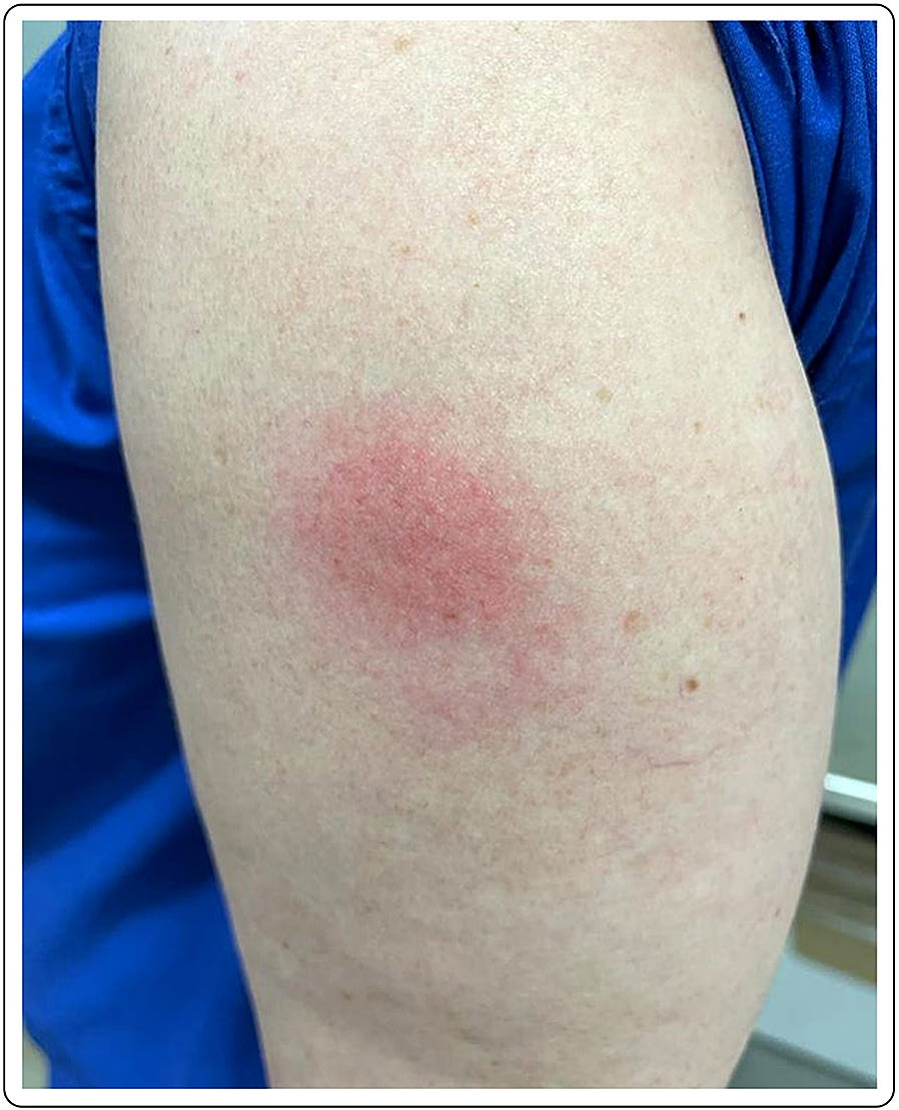
Erythema and edema accompanied by soreness of the arm 7 days after injection of the first dose of the mRNA-1273vaccine

41-year-old Caucasian female with erythema, edema and soreness of the arm presenting 7 days after the first dose of the mRNA-1273 vaccine. Symptoms resolved spontaneously without therapy (Fig. 3).

### Case 4

**Fig. 4:**
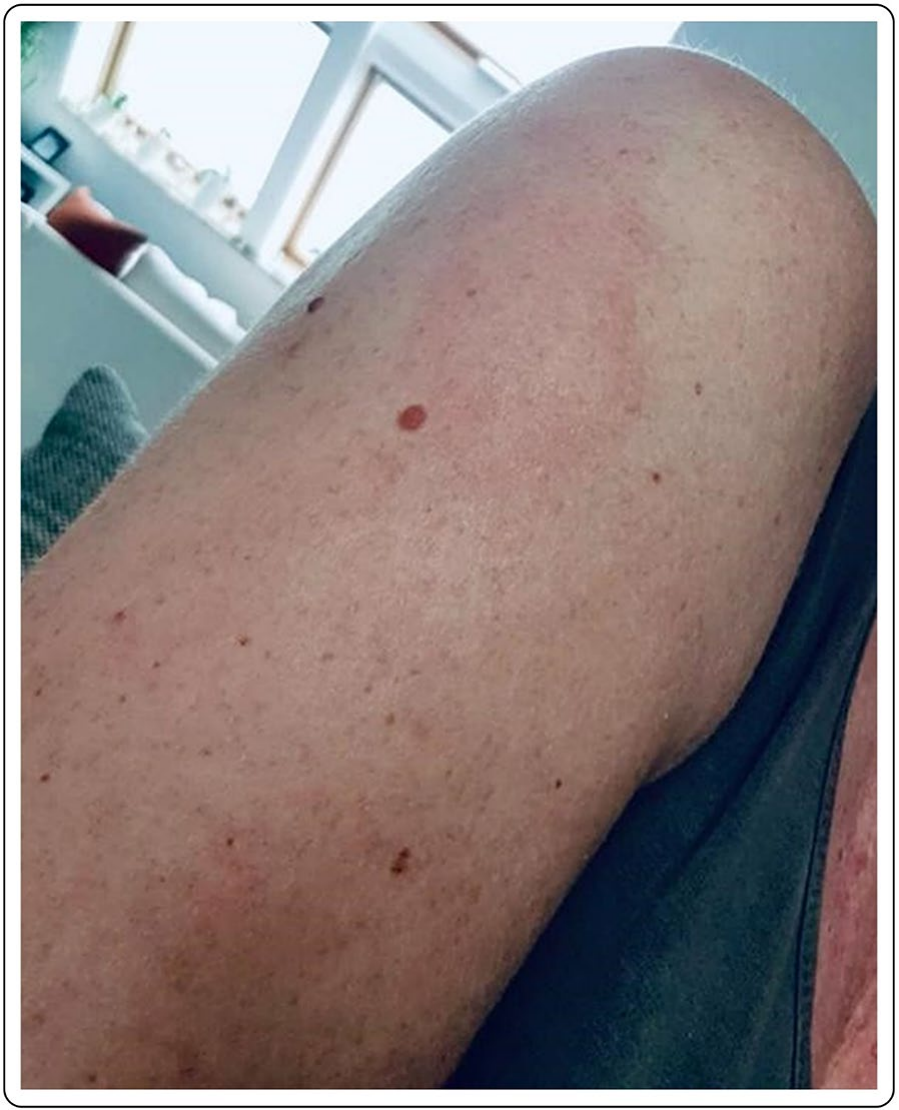
Local erythema at 9 days after the first dose of the mRNA-1273 vaccination

Mild erythema in a 41-year-old Caucasian female 7 days after first dose with the mRNA-1273 vaccine. Complete resolution of the symptoms occurred without treatment after 2–3 days from the onset (Fig. 4).

### Case 5

**Fig. 5:**
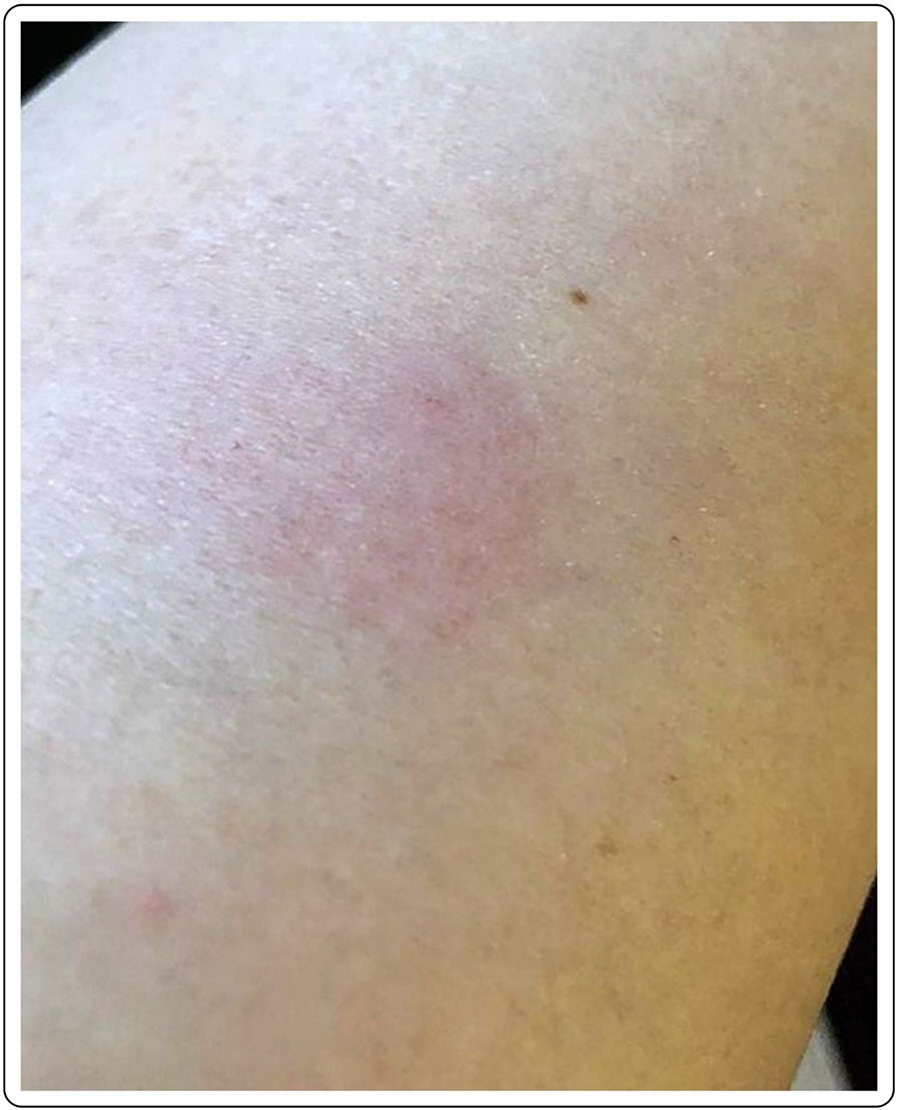
Mild erythema in a 41-year-old Caucasian female 7 days after the first dose of the mRNA-1273 vaccine

50-year-old female with local erythema and soreness of the injected site after the first dose of the mRNA-1273 vaccine. The symptoms appeared 9 days after the injection and disappeared after 2–3 days, without any treatment (Fig. 5).

### Case 6

**Fig. 6:**
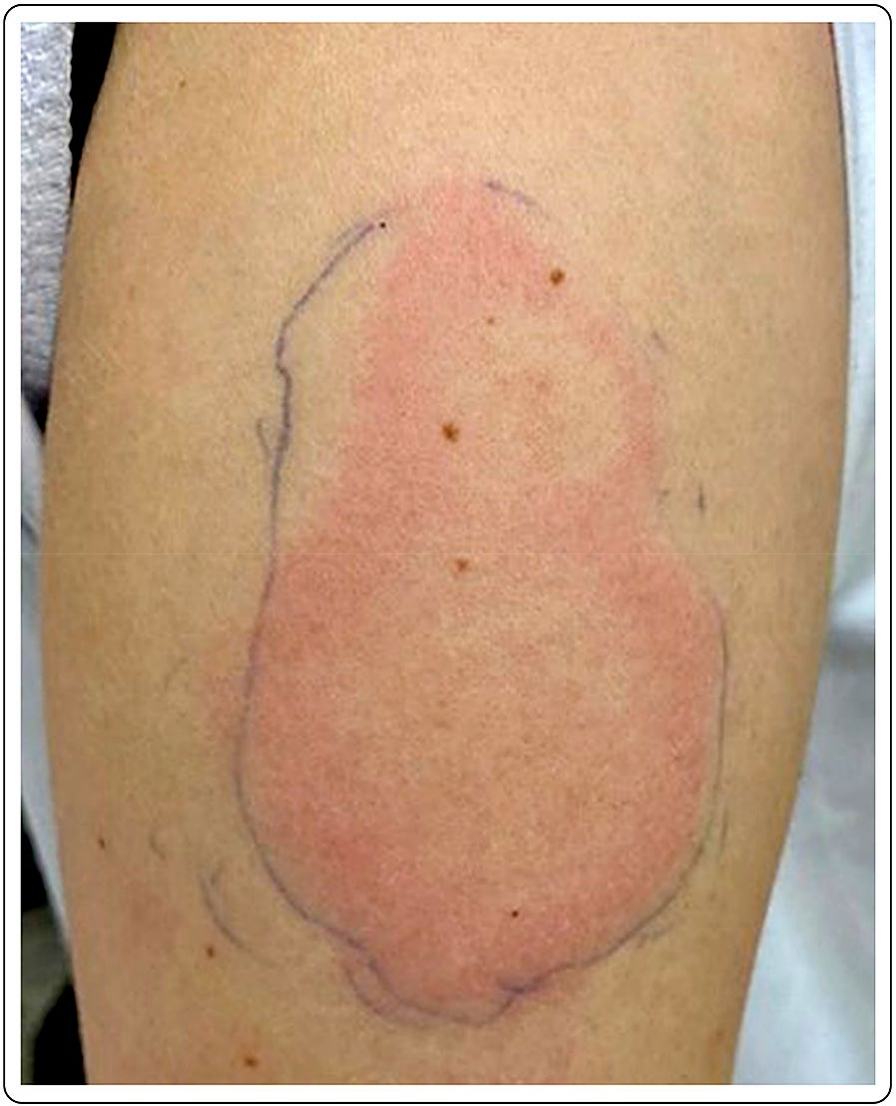
Erythematous and edematous indurated plaque 7 days after the first dose of the mRNA-1273 vaccine

A healthy 30-year-old female developed an 8 × 5 cm indurated plaque on her right arm, 7 days after her first dose of the mRNA-1273 vaccine, which presented as a painful burning sensation at the injection site. She denied any additional systemic or local side effects. Topical methylprednisolone acetate and loratadine orally were prescribed for few days to relieve the discomfort at the injection site. The skin lesions completely resolved after few days. The second vaccination was administered as planned, with no additional acute side effects (Fig. 6).

### Case 7

**Fig. 7:**
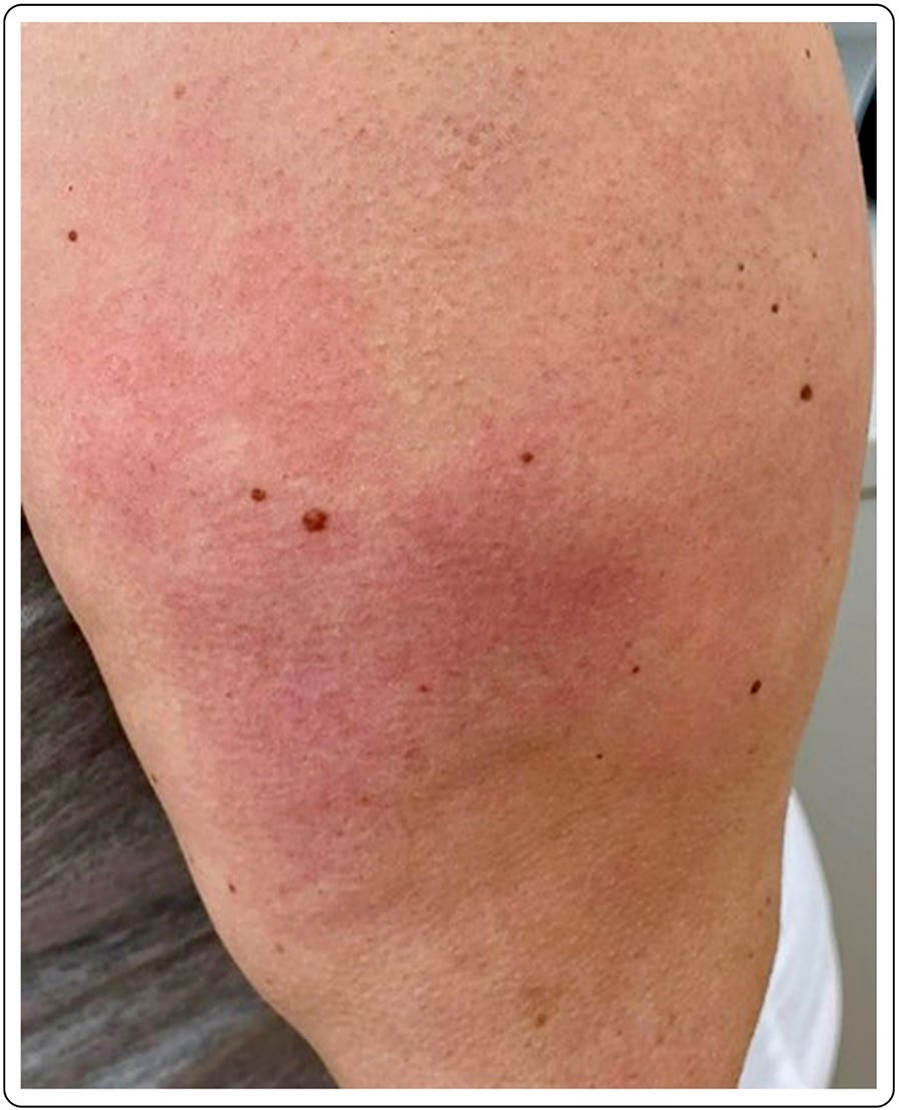
Local erythema and edema 3 days after the second dose of the mRNA-1273 vaccine

A healthy 44-year-old female developed local edema, erythema and induration at the injection site and the surrounding area, 3 days after the second dose of the mRNA-1273 vaccine. No other signs or symptoms were described except for cutaneous tenderness, mild pruritus and chills 24 h after each vaccination. Otherwise, the vaccination was well tolerated. No topical or systemic treatment was needed. There was a complete resolution of the skin lesions after few days (Fig. 7).

### Case 8

**Fig. 8:**
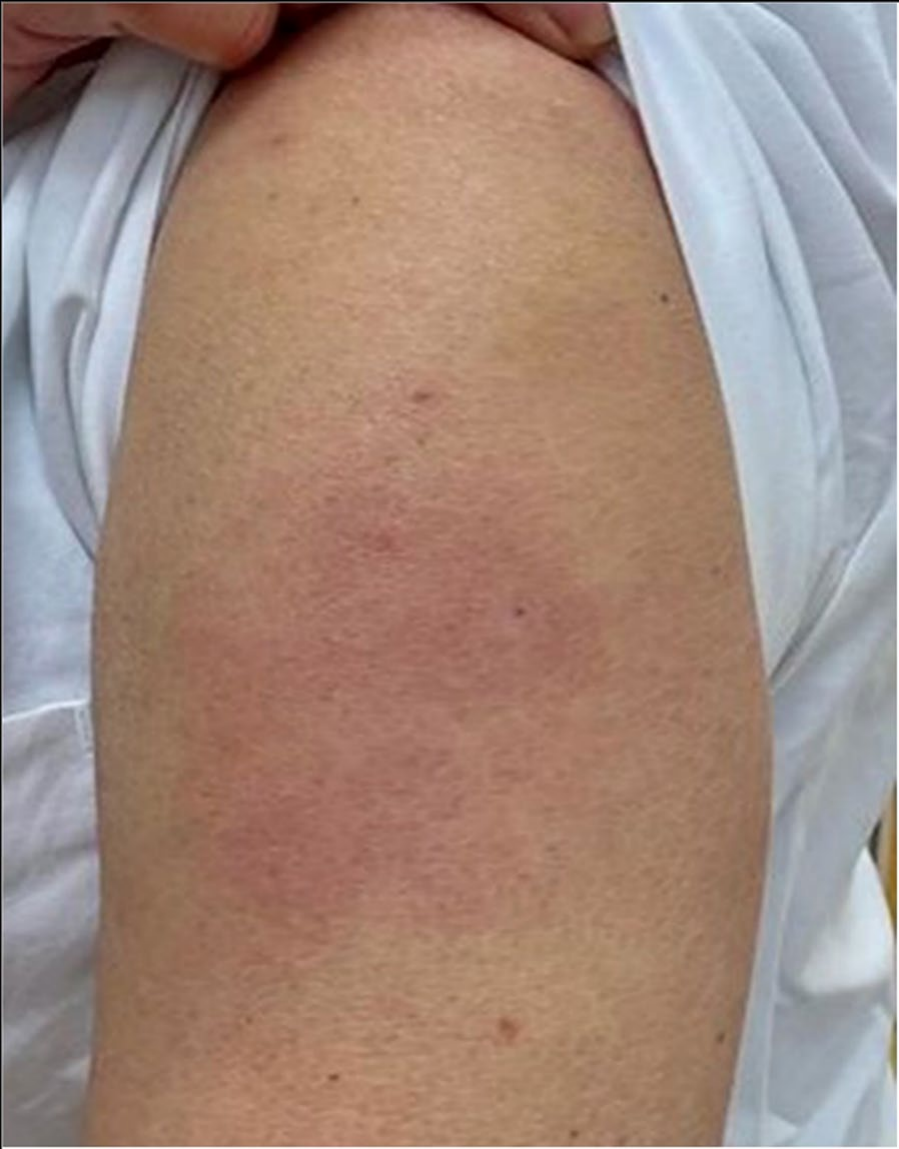
Erythema and edema at the injection site, 2 days after the second dose of the mRNA-1273 vaccine

A 63-year-old female noticed erythema and edema at the injection site on the left arm 2 days after the second dose of the mRNA-1273 vaccine. No other symptoms were described, except for mild pruritus. No known allergies were reported. After the first vaccine dose, the patient felt very tired, and “ill”, requiring her to stay at home. Due to the mild symptoms, no treatment was deemed necessary after the administration of either dose. The cutaneous symptoms spontaneously resolved after 2 days (Fig. 8).

### Case 9

**Fig. 9:**
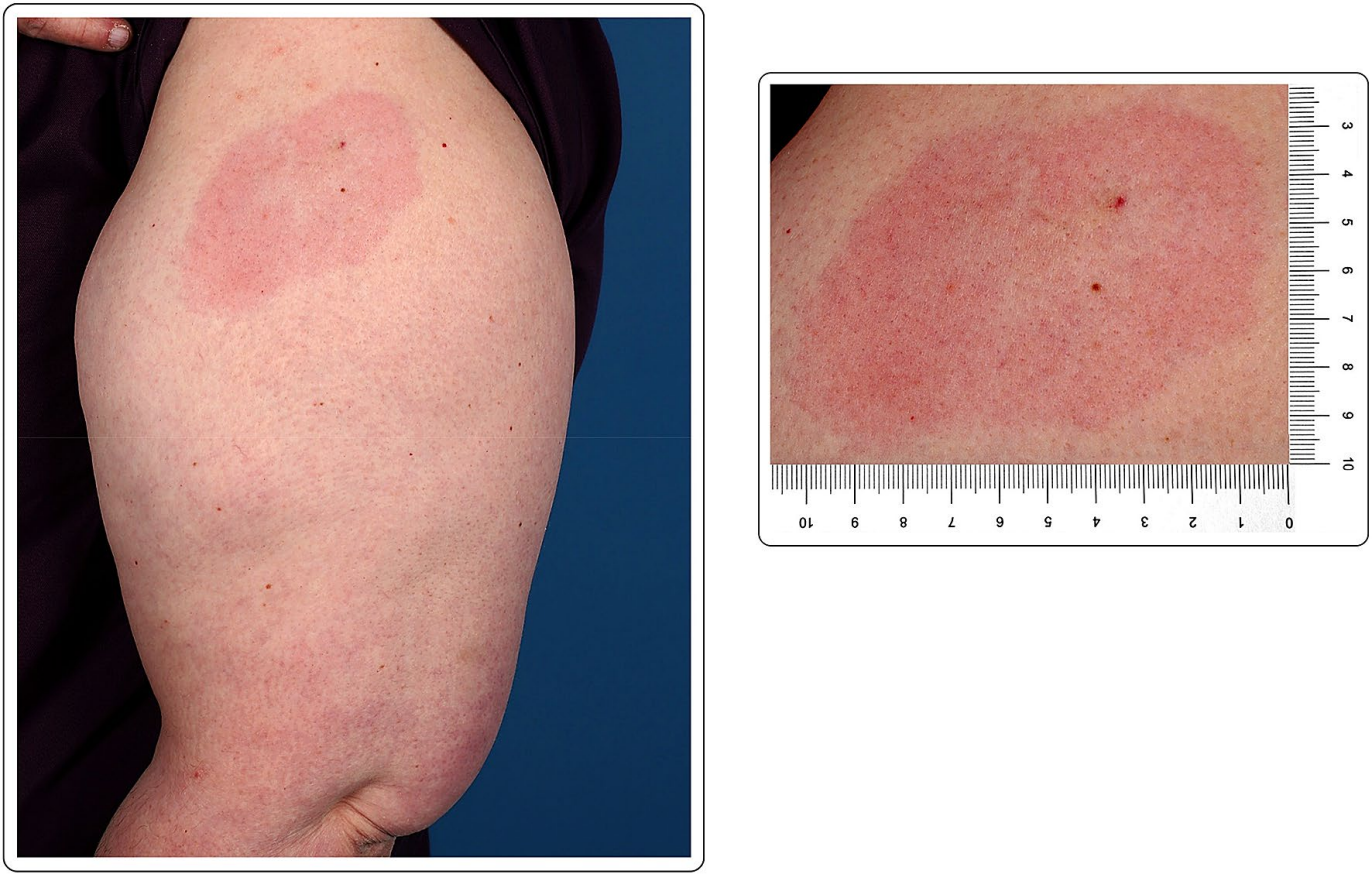
**A** and **B** Erythema, edema and induration 4 days after second dose of the mRNA-1273 vaccine

**Fig. 10:**
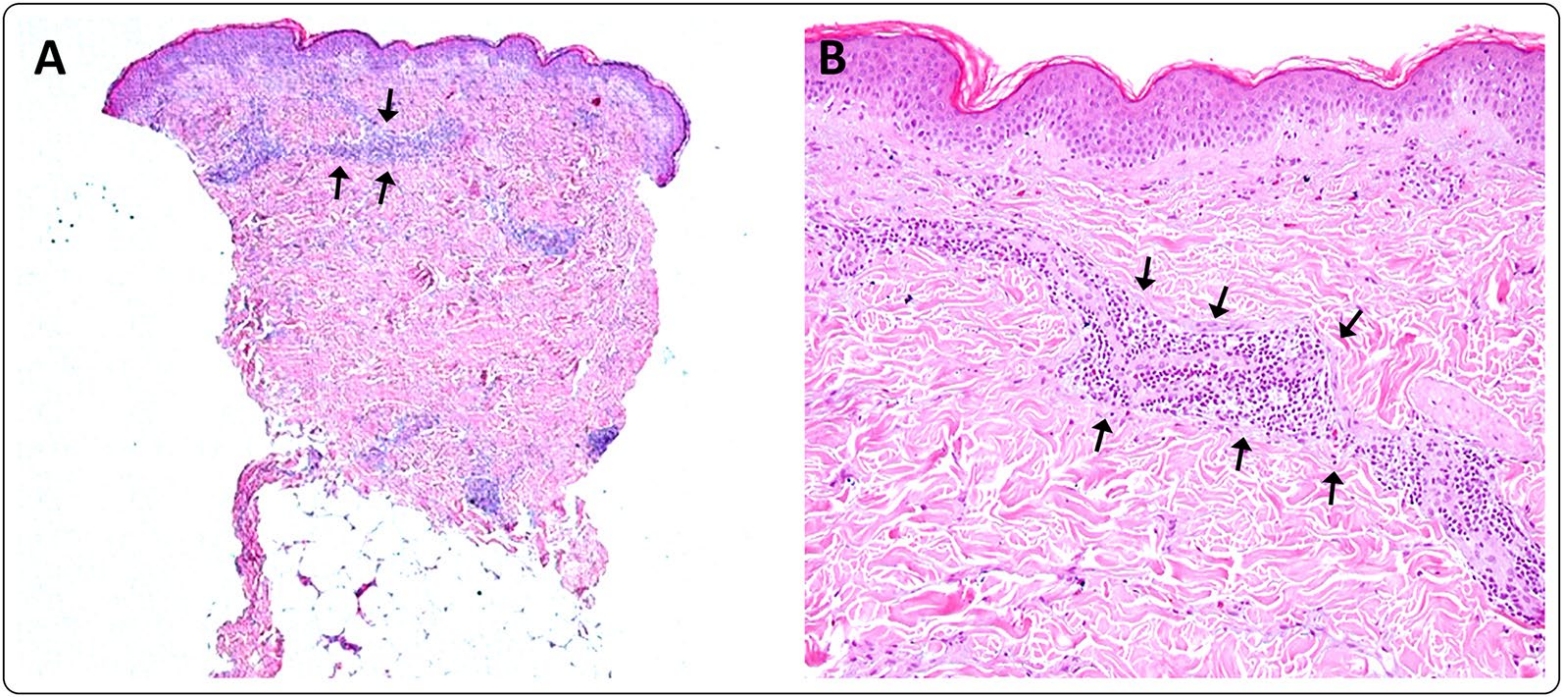
Histology of the injections site of patient 9, 4 days after vaccination. **A**, shows superficial and deep perivascular inflammatory infiltrate in the dermis. The perivascular infiltrate was dominated by lymphocytes (**B**, arrows) [staining: hematoxylin–eosin (HE); original magnification: **A** 40×; **B** 200×]

A 50-year-old healthy female presented with erythema, edema and induration at the injection site, 4 days after the second dose of the mRNA-1273 vaccine (Fig. 9). Due to the significant size of the lesion as well as the pronounced local burning and pruritus, a skin biopsy was taken (Fig. 10).

The histopathologic examination confirmed the diagnosis of a lymphocyte-triggered inflammatory reaction in response to the vaccination with mRNA-1273. The histologic exam revealed a dermal perivascular infiltrate of lymphocytes, and few eosinophils. The patient received topical methylprednisolone acetate until resolution of the skin lesions and loratadine orally for the pruritic symptoms.

### Case 10

**Fig. 11:**
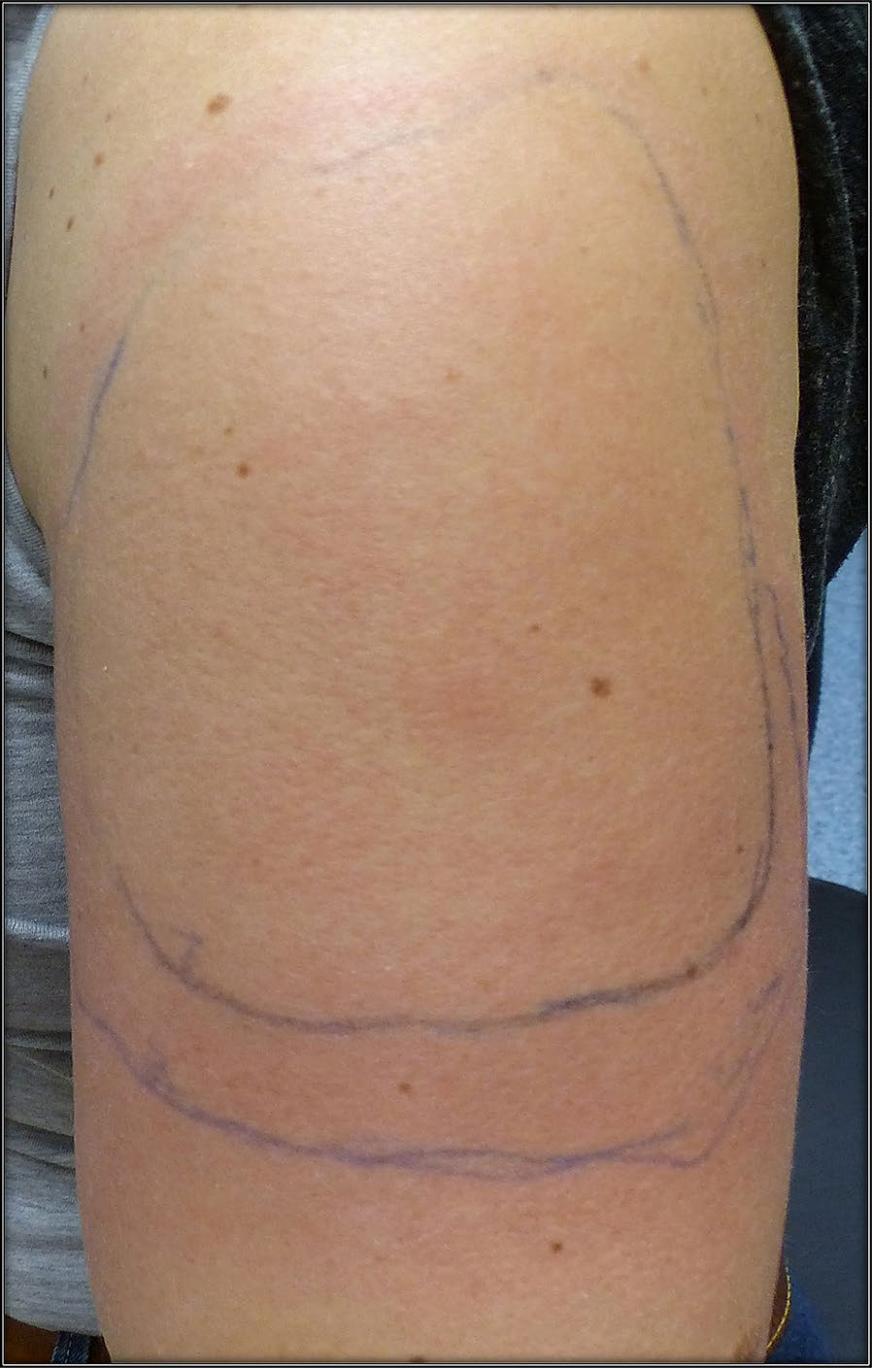
Erythema, edema and induration 8 days after the second vaccination with mRNA-1273

A 37-year old female patient reported painful edema on her left arm, starting 8 days after the first injection with the mRNA-1273 vaccine. A 10 cm-diameter urticarial plaque with central fading was observed. A painful lymph node could also be palpated in the left axilla. The patient felt tired but had no fever (Fig. 11).

### Case 11

**Fig. 12:**
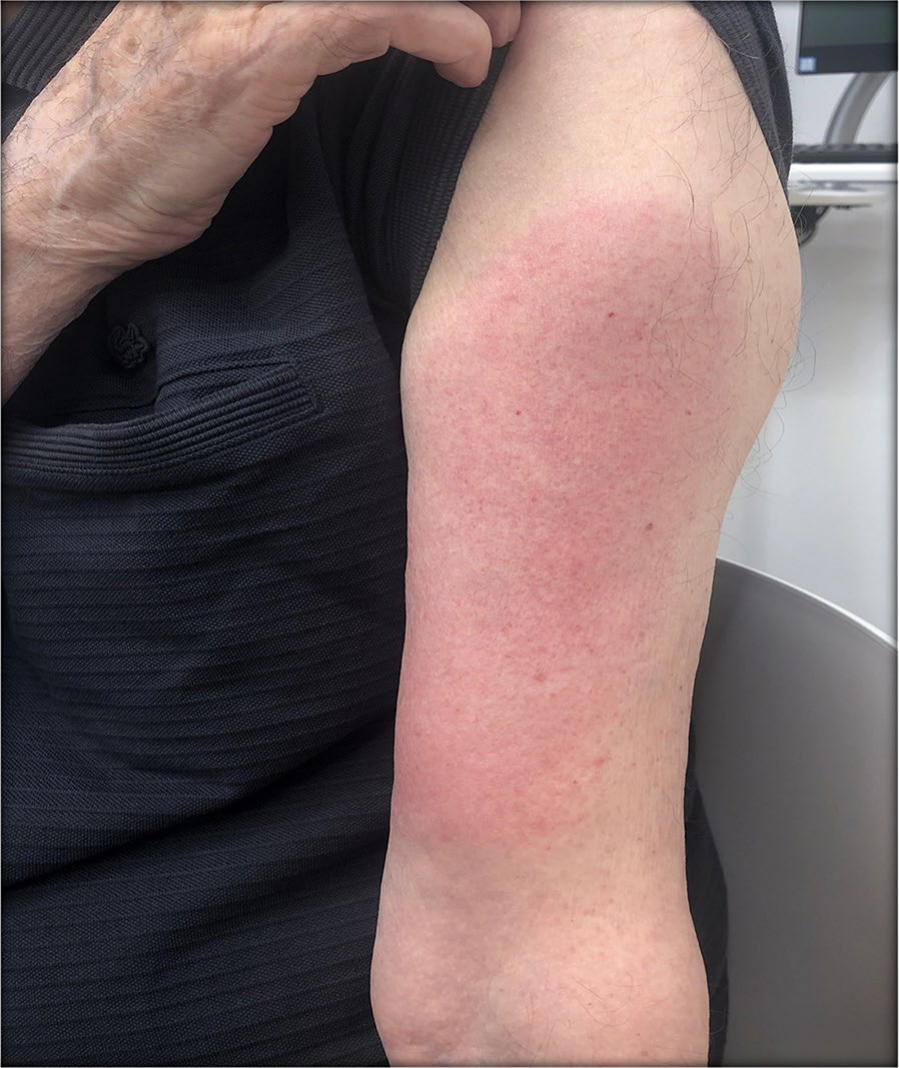
Erythema, edema and induration, 12 days after second vaccination with mRNA-1273

A 79-year-old male patient reported painful edema on his left arm, starting 12 days after the first injection with mRNA-1273 vaccine (Fig. 12).

## Discussion

Our case series of delayed local skin reactions after the mRNA-1273 vaccination is consistent with the recently published literature, reporting similar reactions after the administration of either mRNA-1273 [3, 9] or BNT162b2 [6]; [7] approved vaccinations [6, 7, 9–20]. While the exact mechanism of these skin reactions is still unclear, a delayed hypersensitivity reaction has been hypothesized [11].

Delayed skin reactions are rare post-vaccination events, typically observed several days after the vaccination with both mRNA-based COVID-19 vaccines. The reported incidence is of 0.8–1.0% following the first and 0.2–1.1% after the second dose [2, 6]. These lesions differ from acute allergic and other immediate local reactions attributable to the vaccination itself. The phenomenon is transient and typically resolves within 3–5 days, frequently without any treatment required. In case pharmacological intervention is needed, topical glucocorticosteroids and oral antihistamines are associated with a good response, as highlighted from this study as well as earlier reports.

When the “*COVID arm*” occurs after the first of the two scheduled vaccinations, the recommendation is to proceed with the second dose as planned, administering it to the opposite arm if needed [7, 8].

Currently, it is still unclear why the “COVID arm” occurs after mRNA vaccinations. The delayed skin reactions observed in our case series presented at two distinct times of onset and with two specific clinical phenotypes, indicative of more than a single mechanism of action. Specifically, we observed manifestations of early onset, 2–3 days post-vaccination characterized by diffuse, poorly demarcated urticarial eruption associated with variable degrees of local edema, tenderness, and pruritus well-responsive to antihistamines (as in case #1). These early skin manifestations appeared to be distinct from the ones of later onset (7–10 days after the first dose, and 2–4 days after the second). The erythema in the late lesions was more sharply demarcated and with irregular morphology around and inside the area. While the initial type of skin manifestation clinically reminds of an acute type I allergic reaction, the one with a later onset seems to suggest the involvement of an adaptive immune response, as suggested by earlier reports. Our data, despite the small patient population, seem to be consistent with earlier reports documenting the relative short time from the second-dose administration and the development of the rash [3]. When we compared the delayed skin reactions after the second dose to the early skin manifestations associated with the COVID-19 infection, we found no clinical correlation.

The histopathological analysis of the skin biopsies of COVID-19-associated cases show a diverse range of morphologies. A consistent histological feature, however, appears to be the presence of prominently dilated blood vessels with edematous endothelial layers, vascular engorgement with erythrocytes and perivascular infiltrates [14]. A proposed mechanism of action suggests a direct viral infection of the endothelial cells. Electron microscopy and polymerase chain reaction (PCR) analyses of the skin lesions have supported this hypothesis [13, 20, 21]. It is known that COVID-19 is associated with a wide clinical spectrum of skin lesions including urticarial, vesicular, vasculitic and chilblain‐like lesions. However, the risk factors and the time frame to develop a specific type of skin lesions during the COVID-19 infection are still unclear [7, 15, 22].

The histology of one of our patients after vaccination shows a superficial and deep perivascular dermatitis, with scattered eosinophils and intraluminal neutrophil accumulation (Fig. 10). These features are consistent with previously reported histological analysis of skin lesions following mRNA-based BNT162b2 vaccinations [6].This is similar to the cutaneous histomorphology observed in COVID-19 infections.

We postulate that the skin reactions secondary to mRNA vaccinations belong to a nonspecific histologic pattern referred to as dermal hypersensitivity reaction (DHR) [12]. DHR is not diagnostic for any specific condition or etiology. It is most commonly seen in patients with a urticaria, drug reactions and spongiotic (eczematous) dermatitis. The clinical manifestation of these conditions is similar to the “*COVID-arm*”. Our cases seem to suggest a similarity in the immunologic responses following vaccination. However, additional work is required to further dissect the phenomenon and reveal the underlying immunologic mechanism.

## Conclusion

Delayed local skin reactions, also referred to as “COVID arm”, are a rare side effect that can present as a localized, transient, erythematous and edematous plaque several days after the first or the second dose of the mRNA-based COVID-19 vaccines. Topical glucocorticosteroids and oral antihistamines are effective in resolving the skin lesions and controlling symptoms, even though most cases resolve spontaneously. Patients should be notified that a “COVID arm” is a non-threatening benign potential side effect of the vaccination and it should not discourage from obtaining a second dose of mRNA-based vaccine. Further investigations on the precise molecular and cellular mechanisms underlying this cutaneous pathology are needed to understand why and when rare adverse events may occur afterRNA vaccines.

## Data Availability

All data and materials can be accessed via CM and NH.

## Abbreviations

Covid-19: Coronavirus disease 2019
CDC: Centers of disease control
DNA: Deoxyribonucleic acid
mRNA: Messenger ribonucleic acid
SARS-CoV-2: Severe acute respiratory syndrome coronavirus-2
PEG: Polyethylene glycol
PO: Per os
